# Hagen Bræ: A Surging Glacier in North Greenland—35 Years of Observations

**DOI:** 10.1029/2019GL085802

**Published:** 2020-03-13

**Authors:** A. M. Solgaard, S. B. Simonsen, A. Grinsted, R. Mottram, N. B. Karlsson, K. Hansen, A. Kusk, L. S. Sørensen

**Affiliations:** ^1^ The Department of Glaciology and Climate The Geological Survey of Denmark and Greenland (GEUS) Copenhagen Denmark; ^2^ National Space Institute Technical University of Denmark Lyngby Denmark; ^3^ Physics of Ice, Climate, and Earth Niels Bohr Institute, University of Copenhagen Copenhagen Denmark; ^4^ Danish Meteorological Institute (DMI) Copenhagen Denmark

**Keywords:** glacier surge, ice dynamics, ice flow, North Greenland, remote sensing

## Abstract

We use remotely sensed ice velocities in combination with observations of surface elevation and glacier area change to investigate the dynamics of Hagen Bræ, North Greenland in high detail over the last 35 years. From our data, we can establish for the first time that Hagen Bræ is a surge‐type glacier with characteristics of both Alaskan‐ and Svalbard‐type surging glaciers. We argue that the observed surge was preconditioned by the glacier geometry and triggered by englacially stored meltwater. At present, the glacier is in a transitional state between active and quiescence phases and is not building up to its pre‐surge geometry. We suggest that the glacier is adjusting to the loss of its floating section, general thinning, and changes in fjord conditions that occurred over the study period which are unrelated to the surge behavior. The high temporal resolution of the ice velocity data gives insight to the sub‐annual glacier flow.

## Key Points

- Thirty‐five years of remotely sensed data document the recent dynamics and surge cycle of Hagen Bræ in high detail
- We argue that the surge was preconditioned by geometry but triggered by englacially stored meltwater
- The onset of the observed surge was abrupt while its termination was gradual

## 1 Introduction

The outlet glaciers of North Greenland drain 40% of the Greenland Ice Sheet (Hill et al., 2017). At present, discharge from this region is low relative to other sectors due to the slow flow of its marine‐terminating glaciers (Mouginot et al., 2019; Mankoff et al., 2019). North Greenland glaciers have experienced a general pattern of retreat since the early 1900s, and this trend has accelerated over the past decades (Murray et al., 2015; Hill et al., 2017, 2018). Additionally, several of the marine‐terminating glaciers have lost all or a significant part of their floating tongue (Hill et al., 2018; Mouginot et al., 2015). Superposed on the general trend of glacier retreat, a clear variability in timing and magnitude of retreat is observed. Of the 21 North Greenland marine‐terminating glaciers, eight are classified as being or likely being of surge type—including Hagen Bræ (Hill et al., 2017). Surge‐type glaciers can mask out a response to climatic changes and dramatically modulate discharge on the shorter term due to their surge behavior and make interpretation of the regional dynamics complex (e.g., Yasuda & Furuya, 2015). However, only little is known about surge‐type glaciers in North Greenland, and there is a clear need for glacier‐specific studies in order to understand the underlying mechanisms causing the observed variability (Hill et al., 2017).

A surge‐type glacier is a glacier with periodic or quasi‐periodic fluctuations in ice flow velocity driven by internal mechanisms rather than external forcings (e.g., Benn et al., 2019, Meier & Post, 1969, Sevestre & Benn, 2015). Surge‐type glaciers have longer periods of slow ice flow (“quiescent phase”) interrupted by shorter periods where velocities typically are at least an order of magnitude higher (“active phase”). During the quiescent phase, the low velocities lead to an imbalance in mass flux causing a build‐up of mass and a steepening of the surface slope. At the onset of a surge, the ice flow increases causing the angle of the slope to decrease and the glacier front to advance (Meier & Post, 1969).

Surge‐type glaciers are often categorized as either a Svalbard or an Alaskan type (Kamb et al., 1985; Murray et al., 2003). Alaskan‐type surges (e.g., Variegated Glacier in Alaska) initiate abruptly over winter and have a short active phase (1–3 years). The sudden onset of the active phase is ascribed to a switch in the hydrological system (Kamb et al., 1985; Kamb, 1987; Raymond, 1987). In contrast, Svalbard‐type surges are thermally regulated at the glacier bed (Fowler et al., 2001; Murray et al., 2003), and the active phase initiates with a long period of slow acceleration followed by a shorter period of faster speedup (months). The active phase typically lasts 2–10 years (Dowdeswell et al., 1991) with a long period of slow down (Dowdeswell et al., 1991; Murray et al., 2003). In Greenland both types are found: Surging glaciers in East Greenland and Harald Moltke Bræ, Northwest Greenland were identified by Jiskoot et al. (2003) and Hill et al. (2018), respectively, as Alaskan type while Storstrømmen Glacier and Bistrup Bræ show characteristics of Svalbard type (Mouginot et al., 2018). During both phases of a surge cycle, the flow may be modulated by the seasonal cycle or short‐term melt events (e.g., Flowers et al., 2016; Frappé & Clarke, 2007; Mansell et al., 2012; Yasuda & Furuya, 2015). On longer timescales climatic changes can significantly modify the surge cycle as projected by Mouginot et al. (2018) for Storstrømmen Glacier and by Hill et al. (2018) for Harald Molteke Bræ or terminate the surge behavior all together (e.g., Dowdeswell et al., 1995).

In this study, we document the most recent surge of Hagen Bræ, North Greenland and report on the evolution of the glacier over the past 35 years in high detail. We combine satellite observations of ice velocity from both SAR (synthetic aperture radar) and optical imagery with altimetry data and observations of glacier front position, in order to map the sub‐annual flow over the surge cycle. This provides insights into the processes governing the glacier flow. We investigate the relationship between the surge mechanism and the local climate (modeled by a regional climate model [RCM]) and discuss how the observed changes in glacier geometry and fjord setting can influence the future evolution of the glacier. Understanding the interplay between external conditions and the cyclic nature of surge‐type glaciers is key for interpreting the observed variability in North Greenland glaciers.

## 2 Study Site

**Figure 1:**
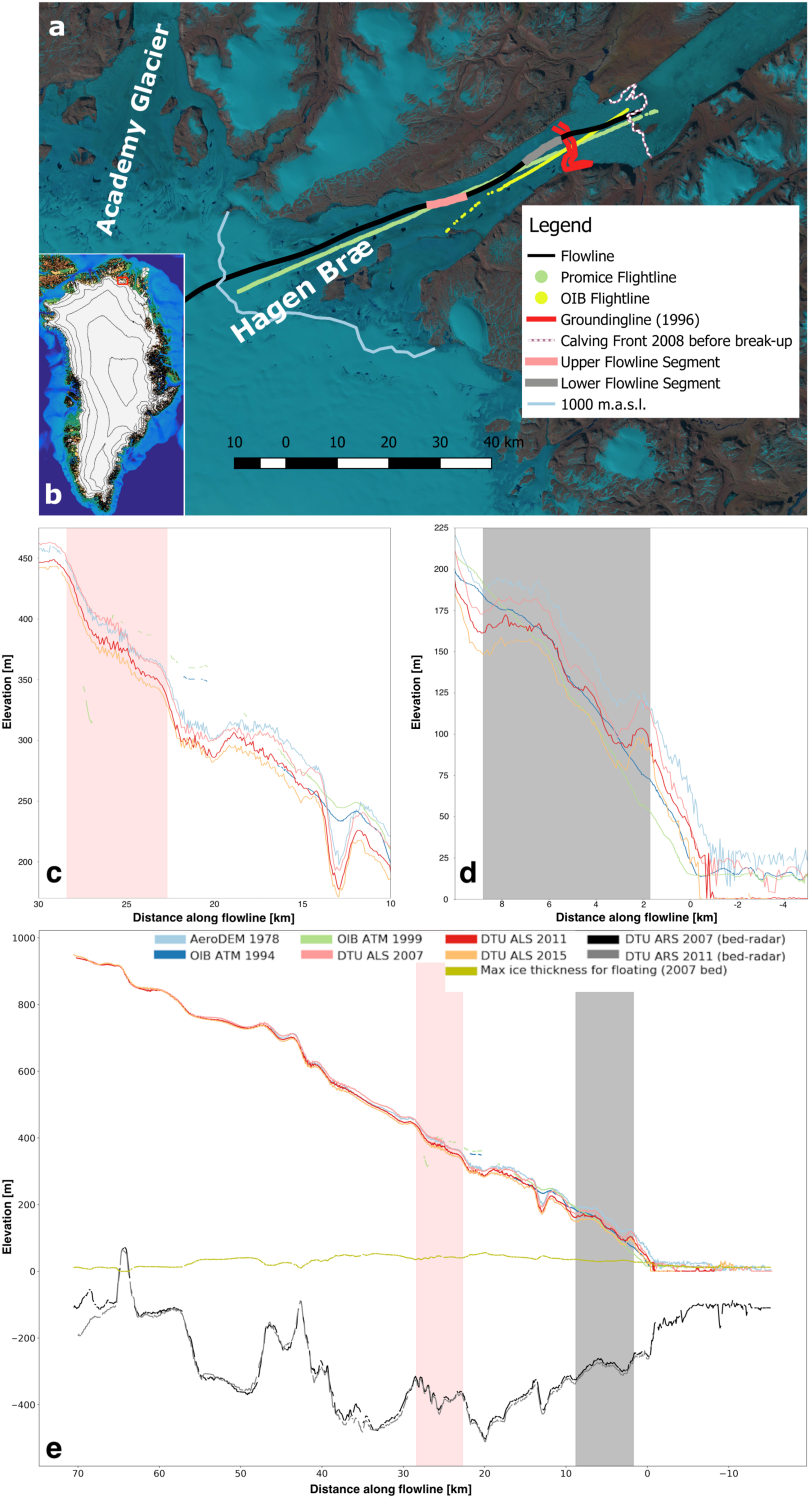
(a) Overview of Hagen Bræ (image from Landsat 5, 27 July 2006). The flowline and the flowline segments used for the ice velocities in Figures 2a and 2c are shown in black, light red, and gray. The grounding line is for 1996 from ESA Greenland Ice Sheet CCI derived from SAR Interferometry. (b) Location of Hagen Bræ in Greenland. (c–e) Surface and bed elevation (bedrock elevation for grounded ice and base of glacier downstream of grounding line) data along the flightlines indicated in (a) from DTU/Promice ALS (2007, 2011, and 2015) and from OIB (1994 and 1999). The 1978 AeroDEM surface elevation interpolated along the DTU/Promice‐flightline is also shown. (c) and (d) are zoom‐ins.

Hagen Bræ drains 6% of North Greenland by area (Hill et al., 2018) making it one of the major outlets in the region (see Figure 1a). The glacier is 75 km long and approximately 10 km wide and flows into Hagen Fjord—a side fjord to Independence Fjord (Higgins, 1990). The glacier is situated on bedrock below sea level, and this subglacial trough extends more than 100 km inland and connects to the neighboring Academy Glacier (Morlighem et al., 2017). No reports exist of a surge at Hagen Bræ, but several studies have hypothesized that it is a surge‐type glacier (Abdalati et al., 2001; Csatho et al., 2014; Hill et al., 2017; Rignot et al., 2001; Thomas et al., 2009). Snapshots of the flow and geometry of the glacier back in time indicate changes in flow: Davies and Krinsley (1962) report that “Practically the entire glacier is now stagnant.” from field observations during the last half of the 1950s, whereas Higgins (1990) provides average values of flow between 1947 and 1978 close to the calving front of $1.4\;m\;{\text{day}}^{-1}$ based on 30 years of photographic coverage. Observations of the frontal extent of Hagen Bræ date back to the beginning of the 20th century due to the cartographers H. Hagen (Danmarks Ekspeditionen) and L. Koch (2. Thule Expedition). Later observations include aerial photographs from the 1950s, 1960s, and 1970s (Davies & Krinsley, 1962; Higgins, 1990) and satellite imagery from the 1980s and forward. All accounts suggest that a floating tongue has been a persistent feature of the glacier over the past 100 years until summer 2008 when the tongue broke up and the glacier retreated 15 km (Hill et al., 2017).

## 3 Data and Methods

In this study we rely on multiple data sets on different temporal scales:

- Ice flow velocities from 1985 to present day.
- Airborne altimetry data.
- Digital elevation models of the ice surface from 1978 to present day.
- Observations of glacier area change from satellite spanning 1985 to present day.
- Bedrock topography from ice‐penetrating radar.

In addition we rely on the results from an RCM covering 1985 to 2016. Below, we present each data set in more detail.

The ice velocity (IV) maps are derived from satellite SAR and optical data. The SAR‐based maps are freely available through Programme for Monitoring of the Greenland Ice Sheet (PROMICE) (www.promice.org) and the ESA (European Space Agency) Climate Change Initiative Greenland Ice Sheet projects (www.esa-icesheets-greenland-cci.org). The maps span 1991 to 2010 and 2015 to 2019 and are based on intensity offset tracking of ESA's ERS‐1 and ERS‐2, Envisat, and Sentinel‐1 data (Dall et al., 2015; Kusk et al., 2018; Nagler et al., 2015; Strozzi et al., 2002) and have an uncertainty of 3–10 cm day ${}^{-1}$. We extended the ice velocity data set back to 1985 and filled in the gaps in the SAR record through feature tracking of optical images from the Landsat archive (Messerli & Grinsted, 2015). Based on comparison with stable ground the estimated error is 10 cm day ${}^{-1}$. From 2013 the sub‐seasonal behavior of the glacier can be resolved thanks to the launch of Landsat 8 and Sentinel‐1.

Surface elevation of Hagen Bræ has been measured by the airborne PROMICE campaign (Ahlstrøm & The PROMICE team, 2008; Sørensen et al., 2018) with an uncertainty of <10 cm. In 2007, 2011, and 2015 an airborne laser scanner (ALS) was flown along a central flowline (Forsberg et al., 2001). In addition, three older data sets are included in our analysis: (a) The AeroDEM based on aerial photographs from 1978 to 1987 (Korsgaard et al., 2016). (b) The Operation IceBridge (OIB) Airborne Topographic Mapper (ATM) LiDAR system (Krabill et al., 2009). (c) The sporadic measurements of elevation by the ICESat‐mission 2003–2009 (supporting information Figure S3). For the AeroDEM, the subset of the gridded data that covers Hagen Bræ dates to 1978 and has been interpolated onto the PROMICE flight path. The surface elevation from OIB is available from multiple campaigns (2009) through the National Snow and Ice Data Center (NSIDC). Here, we use the Level 4 product to deduce the observations of Hagen Bræ from 1994 to 1999. The surface elevation profiles are shown in Figures 1c–1e. Note that the OIB data are displayed for the lower part of the glacier only, where the flight paths are close to the PROMICE flight path.

The bedrock topography along the PROMICE flight path (cf. Figure 1e) stems from direct measurements conducted with a 60 MHz coherent ice‐penetrating radar (Christensen et al., 2000) during the PROMICE airborne campaigns.

Glacier area change is obtained from mapped annual changes in the areal extent of Hagen Bræ since late summer 1985 (Jensen et al., 2016). Images from Landsat and Sentinel‐2 were used (see Figure S6). A time series of Landsat images showing the evolution of the outer part of the glacier can be found in Figures S7 and S8.

To elucidate a potential climate signal in the variability of the glacier flow regime, we compared the IV and glacier area change with surface mass budget and runoff data from the HIRHAM5 RCM (Mottram et al., 2017). The RCM has a resolution of ≈5 km and is forced by the ERA‐Interim climate reanalysis for 1979–2017. Langen et al. (2017) showed that the model gives good results for modeling surface mass balance when compared with the observational record, although Northern Greenland has very few observations that can be used for model evaluation.

## 4 Results

The high resolution and long temporal span of the IV data, in combination with observations of surface elevation change and optical satellite imagery from Landsat, unveil a story of dramatic changes in glacier flow and geometry over the past 35 years. Briefly, we suggest that at the beginning of our data series in 1985 the glacier is at the end of a surge with IV up to 2 m day ${}^{-1}$. During this period, the floating section extended well beyond the islands in the fjord (cf. Figure 1). The northern branch of the glacier advanced between 1985 and 1988 but retreated between late summer 1988 and 1990 concurrent with a slowdown of glacier flow to <0.5 m day ${}^{-1}$. This was interrupted by a surge starting in 2002, where surface velocities peaked at almost 3 m day ${}^{-1}$ and the winter velocities were consistently high ( >1 m day ${}^{-1}$ at the front). Since approximately 2012, the glacier has exhibited low winter velocities ( <1 m day ${}^{-1}$ at the front) and a distinct summer speedup upward of 2 m day ${}^{-1}$. The overall pattern of surface elevation change is one of general thinning of at least the outer 40 km of the glacier between 1978 and 2015.

**Figure 2:**
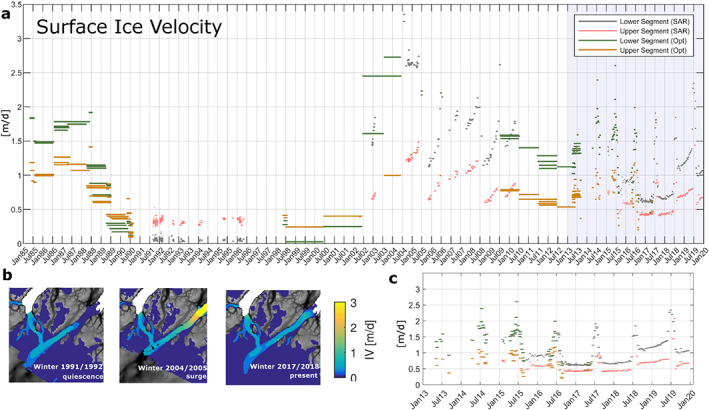
(a) Ice velocity time series. The average velocity of the two flow line segments for each velocity map (for location see Figure 1a). The width of each bar shows the time span between the acquisitions. (b) Map plan view of the ice flow averaged over winter for three winters. (c) Zoom in on the shaded area in (a).

Figure 2a shows the 1985 to present time series of IV averaged over two segments of the flow line (one close to grounding line and one further inland) indicated in Figure 1. The data set relies solely on Landsat imagery during the 1980s and often the IV maps span more than a year. In 1985, the velocity regime is typical of marine‐terminating glaciers where the fastest flow is at the front. In the late 1980s, the glacier slowed down significantly and entered a new flow regime during 1989: The flowline segment closest to the grounding line slowed to less than 0.2 m day ${}^{-1}$ while the upper segment also slowed but to a lesser extent (gray and red areas on Figure 1a, respectively), thereby reversing the earlier IV pattern. The slowest moving part of the glacier was located 5 km upstream of the grounding line (Figures S1 and S2) and moving at a pace of centimeters per day approximating deformational rates (no sliding). The velocity progressively increased to ≈0.6 m day ${}^{-1}$ approximately 40 km inland and also increased slightly toward the grounding line.

This quiescent phase persisted throughout the 1990s with very little variability in magnitude of IV and glacier extent. Note that IV data for this period are mainly SAR‐derived maps with a heavy over‐representation of winter values. Two pairs of optical images in the summer of 1998 suggest that the outer part of the glacier accelerated in summer. During this phase, surface elevation increased upstream of ≈8 km from the 1996 grounding line and decreased downstream leading to an increase in the slope of the glacier (Figures S4 and S5). Between 1994 and 1999, the slope increased 6–7 km upstream of the grounding line by ∼0.2 °, while the slope had increased ∼0.5 ° between 1978 and 1999. Overall, Hagen Bræ has a low surface slope generally less than 1 °.

The quiescent phase ended abruptly during or following the summer of 2002 when the velocity increased by approximately an order of magnitude and a surge followed (Figure 2a). The northern branch of the floating tongue pushed forward by ≈3 km between the end of summer 2002 and the end of summer 2007. The fastest flow was in the area closest to the grounding line. One IV map spanning the period summer 2000 to summer 2002 suggests that both segments accelerated slightly prior to the main speedup. During the surge, the glacier speedup over the winter, and the velocity peaked in summertime and slowed down at the end of the melt season—a pattern that extended at least 40 km upstream of the grounding line. The fastest velocities are observed between 2003 and 2005. Hereafter, the velocities decreased.

The surface elevation measurements from ICESat (Figure S3) covering the period 2004–2009 are noisy but show the classic behavior of a glacier during a surge: general thinning upstream and general thickening on the outer ≈10 km. In summer 2008, the floating tongue broke up, but no acceleration followed this event. Since 2008, the glacier has retreated further every year except in 2011 where it advanced slightly.

Following the deceleration, the main speedup occurs in summer except for 2018/2019 where speedup started over winter and peaked the following summer. We observe two types of flow behavior characterized as Type 2 and Type 3 by Moon et al. (2014). Type 2 glaciers have a strong correlation in the timing of peak flow and peak surface melt whereas Type 3 exhibits a slow down at peak surface melt and a clear midsummer minimum indicating a transition from an inefficient to an efficient subglacial drainage system. For Hagen Bræ, a midsummer minimum is observed in 2015 and 2016 (and less confidently in 2013). Surface elevation measurements from 2007, 2011, and 2015 show that the glacier thinned on the outer ∼40 km with the largest changes occurring in the first interval. Inland, 40 km from the 1996 grounding line, surface elevation changes have been minor.

**Figure 3:**
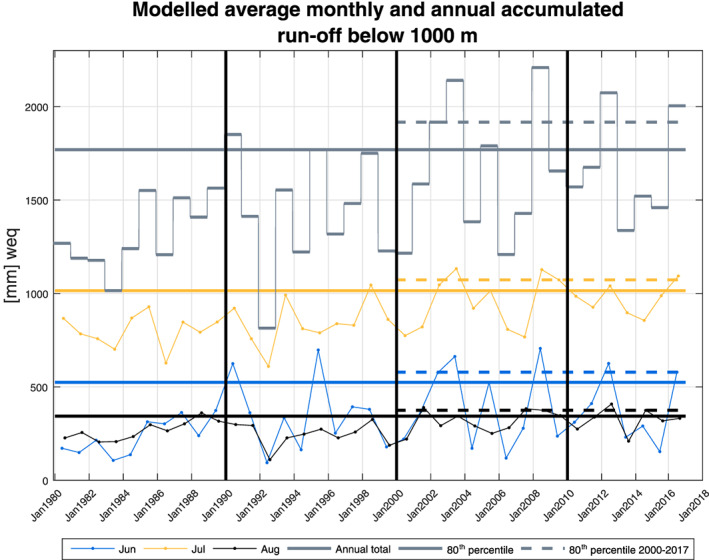
Modeled average surface runoff below the 1,000 m contour using HIRHAM5: Annual and monthly values for June, July, and August are shown together with the 80th percentile both for the entire period as well as the period since 2000.

Figure 3 shows the average surface runoff on the glacier below the 1,000 m elevation contour (Figure 1a) as modeled by the RCM. Annual results are displayed along with the results for the summer months June, July, and August. In 2002 (the year of the onset of the surge) the runoff was particularly high for both July and August as well as the annual total (above the 80% percentile for 1980–2016). In general, runoff increases over the period, and following 2002 several years had high runoff, peaking in 2008 the year in which the shelf broke up.

## 5 Discussion

The combined observations of ice velocity, surface elevation, and glacier area change document in high detail the surge of Hagen Bræ starting during or following the end of the summer of 2002. We argue that our data clearly demonstrate that Hagen Bræ is a surge‐type glacier, and we will expand on this argument below. Further, the high temporal resolution of the IV data since 2013 reveals details in the flow that are not resolved earlier. We discuss the present state of Hagen Bræ and implications for its future evolution.

The first characteristic of a surge‐type glacier stated by Meier and Post (1969) is that “All surging glaciers surge repeatedly.” There are no reports of surges of Hagen Bræ prior to the one documented in our data set but the snapshots of ice velocity back in time combined with our velocity and glacier area change record extending back to the 1980s substantiate the case for previous surges. The observations by Davies and Krinsley (1962) suggest that the glacier was in a quiescent phase during the 1950s similar to the 1990s. The average velocity reported by Higgins (1990) for the period 1947–1978 is an order of magnitude higher than the flow during the 1990s but similar to the observations from the 1980s ( ≈2 m day ${}^{-1}$) and slower than during the peak of the surge (up to ≈ 3 m day ${}^{-1}$). In combination with IV during the 1980s which we interpret to be the end of a previous surge, we suggest that a surge started sometime during the 1970s. The high 30‐year average velocities observed by Higgins (1990) also hint at the possibility of a third surge cycle sometime during 1947–1978.

Based on these considerations, we conclude that Hagen Bræ is a surge‐type glacier and that it has both Svalbard and Alaskan type characteristics unlike other known Greenland surge‐type glaciers (Jiskoot et al., 2003; Hill et al., 2017; Mouginot et al., 2018): It has a relatively long average surge cycle period of 20–30 years (based on three or two surge cycles over 60 years (1950s–2010s), respectively). We consider the surge to end between 2010 and 2013 when the deceleration slowed and the glacier started to retreat. Thus the active phase is long based on the recent surge phase which lasted approximately a decade. This is typical of Svalbard‐type behavior including the gradual termination of the surge (both in the case of the recent and previous surge). However, the rapid onset over winter of the active phase is a characteristic attributed to Alaskan type.

The sudden transition to very fast flow and considerable winter speedup indicates a switch in the sub‐glacial drainage system (Harrison & Post, 2003; Kamb, 1987; Raymond, 1987). Lingle and Fatland (2003) propose a mechanism for temperate glaciers concerning englacially stored meltwater as a surge trigger that we consider to be relevant for Hagen Bræ although it is not temperate. Surging at other non‐temperate glaciers have also been suggested to be influenced by input from surface melt (Yasuda & Furuya, 2015). Lingle and Fatland (2003) suggest that some of the surface meltwater produced in late summer/early autumn becomes trapped englacially due to the slow drainage system at this time of year. Gradually over winter this water moves to the bed due to the ice‐water density difference thereby increasing the water pressure in the subglacial system leading to a steady increase in surface velocity. As the glacier builds up to its pre‐surge geometry it thickens and steepens. And as the glacier thickens it is able to progressively store more water internally until it reaches a threshold where the pressure is high enough to trigger a surge (Lingle & Fatland, 2003). However, it is not enough for a glacier to be able to store sufficient water; the meltwater must also be available. This is controlled by meteorological conditions at the surface.

We argue that it was the large amount of surface meltwater produced in the summer of 2002 that kick‐started the surge. Prior to 2002, total runoff was also high in 1990, 1995, and 1998 (see Figure 3). Thus, on its own, a large amount of available meltwater is insufficient to trigger a surge (cf. Lingle & Fatland, 2003). Abe and Furuya (2015) observed winter speedup far from the terminus on Canadian surge‐type glaciers in their quiescent phase. The authors hypothesized that this type of flow can be explained by the mechanism put forward by Lingle and Fatland (2003) and that the glaciers remained quiescent because they had not reached a geometry where a sufficient amount of meltwater is stored in order to initiate a surge. Our observations also document winter speedup outside the active phase in 2018/2019 supporting their hypothesis. In addition, the timing of meltwater input plays a role. Late season melt is more likely to encounter a drainage system that has shut down. In 2001, August runoff was the maximum to date in our timeseries. This late season melt could help elevate internal water pressure prior to the high melt‐input of the following summer providing further preconditioning. We suggest that surge conditions were primed by thickening of the ice, increase in slope, and late season high melt in 2001 but that the timing was determined by the large amount of meltwater produced in summer 2002. A study of two tidewater glaciers in Svalbard also concluded that the observed surges were preconditioned by internal mechanisms but set off by external processes (Sevestre et al., 2018).

Following 2002, runoff was high (above the 80th percentile) in 2003, 2005, and 2008. The fast flow, however, was also sustained in years with low melt indicating that other processes contributed. Although the surge was not triggered by the thermal‐switch mechanism (Fowler et al., 2001; Murray et al., 2003), frictional heating at the bed could help sustain the fast flow by increasing meltwater production and ice temperature.

From 2013 and onward, our observations resolve the sub‐annual flow revealing transitions between Type 2 and Type 3 behavior as classified by Moon et al. (2014) and significant winter speedup. We expect both the timing and amount of available meltwater to be governing factors for switching the drainage system from inefficient to efficient and to determine when sufficient water becomes trapped over winter to result in speedup. However, we observe no correlation between the timing of meltwater input and flow type for the short overlap between IV and RCM output (2013–2016) in this study. We attribute this to the monthly resolution of the RCM data presented here which is too coarse to study the precise timing of transitions in the drainage system relevant for Type 2 and Type 3.

The lack of speedup (Figure 2a) following the loss of the tongue in summer 2008 indicates that by then it no longer provided a buttressing effect. This is unlike Zachariae Isstrøm where acceleration and loss of the ice shelf was observed simultaneously (Mouginot et al., 2015). We ascribe this difference to the surge behavior of Hagen Bræ as discussed in the following: The processes causing the disintegration had been working for some time. Satellite images (Figures S7 and S8) and the 1978 AeroDEM show that the floating tongue had many fractures and apparently was stabilized by the islands and sea ice in the fjord. Thinning of the tongue since 1978 up to its disintegration is documented by the surface elevation measurements (Figures 1c–1e). We attribute this thinning to extremely low ice flux at grounding line during quiescence. We document a transition toward earlier breakup of the fjord ice by approximately a month between the 1980s and the 2000s using Landsat images (Figure S10). This is an indirect indication that the fjord waters became warmer. Rignot et al. (2012) studied the spreading of warm ocean waters around Greenland 1992–2009 using an ocean model. They found a significant increase in subsurface temperatures off the North Greenland shelf over the period substantiating our indirect observations. Mouginot et al. (2015) conclude that ocean warming played a major role in the extensive retreat of Zachariae Isstrøm starting in the early 2000s, a result confirmed by Heuzé et al. (2016) and Muenchow et al. (2016) for Petermann glacier. The combination of a thinner floating tongue, earlier breakup of the fjord ice, warmer waters in the fjord, and increased surface melt most likely preconditioned the floating tongue for breakup. Previous surges did not cause the tongue to disintegrate. Thus, we attribute the breakup of the floating tongue in 2008 to reduced fjord ice, increased ocean temperatures (Rignot et al., 2012), and increased runoff.

It is an open question whether Hagen Bræ has been in an active phase or a quiescent phase since the beginning of 2010. The glacier has thinned and retreated almost every year since 2008, but it has not returned to the stagnant and reversed IV pattern observed in the 1990s. Presently, the IV are slower than during the surge (in comparison with the mid‐1980s and following 2002) and are more comparable to the values during the short (2–3 years) transitional period in the late 1980s leading to the quiescent phase. Due to the general thinning and retreat it could be argued that the glacier is in a similar transitional phase; however, this phase has currently lasted nearly a decade. In other words, a change in surge cycle could be underway analogous to Storstrømmen Glacier where changes in climate are projected to increase the surge cycle length (Mouginot et al., 2018). Precisely how climate change is affecting the surge behavior of Hagen Bræ is not easily resolved, but from our observations, we can surmise that the present day setting is different compared to the end of the previous surge in the 1980s: (a) The glacier has lost its floating tongue and the buttressing it provided on ice flow. The conditions that caused the disintegration prevail making a reappearance unlikely. (b) The glacier is generally retreating (Figure S6), and the outer 20 km has thinned since 1978. (c) Increased surface melt.

At present, Hagen Bræ is resting on bedrock located below sea level at least ∼70 km upstream from the grounding line. Bedrock is above sea level in only a few locations (Figure 1c). This setting makes it vulnerable to exposure to warm ocean water especially since it presently sits on a retrograde slope extending 20 km inland. In Figure 1e the minimum ice thickness for no flotation is plotted, showing that a thinning of the order of $10^{2}$ m is needed for the glacier to start floating and thereby reduce backstress. Based on this we conclude that the glacier is not threatened by immediate collapse although increased surface and sub‐marine melt and grounding line retreat will affect this scenario.

## 6 Conclusions

In this study we document the surge cycle of Hagen Bræ, North Greenland in high detail using 35 years of remotely sensed data. We verify that the glacier share characteristics with both Alaskan‐ and Svalbard‐type surging glaciers. We argue that the surge is related to internal storage of meltwater and that the sudden onset of the surge following summer 2002 was triggered by a high amount of surface melt. The distinct inter‐annual variations in the glacier's seasonal cycle, temporally resolved in our observational data since 2013, are most likely also caused by the timing and magnitude of surface water input.

For the last decade, Hagen Bræ has been in a transitional phase between surge and quiescence. This transitional phase is much longer than the previous one of 2–3 years. We surmise that the glacier is adjusting to changes which occurred over the past decades and which are unrelated to the intrinsic surge dynamics. The changes include earlier breakup of the seasonal fjord‐ice, the loss of the floating tongue in summer 2008, and higher surface melt. It is likely that these factors will influence the coming surge cycles, similarly to the changes in the surge cycle length of Storstrømmen Glacier caused by a change in climate (Mouginot et al., 2018). Thus investigating surge‐type glaciers in a changing climate is important for understanding the mechanisms leading to the observed variability in North Greenland retreat noted by Hill et al. (2017).

## Supporting information



Supporting Information S1
