# Supplementary material for: Hagen Bræ: A Surging Glacier in North Greenland—35 Years of Observations
Source: Geophys Res Lett. 2020 Mar 13;47(6):e2019GL085802. doi: 10.1029/2019GL085802 (PMC7375144; doi:10.1029/2019GL085802)
Supplement: Supplementary file 1 — Supporting Information S1 [file GRL-47-e2019GL085802-s001.pdf]

Supporting Information for
"Hagen Bræ: A surging glacier in North Greenland – 35 years of observations"

A. M. Solgaard[1], S.B. Simonsen[2], A. Grinsted[3], R. Mottram[4], N. B. Karlsson[1], K. Hansen[1], A. Kusk[2] and L. S. Sørensen[2]

[1]The Department of Glaciology and Climate, The Geological Survey of Denmark and Greenland (GEUS), Copenhagen, Denmark

[2]National Space Institute, Technical University of Denmark, Lyngby, Denmark

[3]Physics of Ice, Climate, and Earth, Niels Bohr Institute, University of Copenhagen, Copenhagen, Denmark

[4]Danish Meteorological Institute (DMI), Copenhagen, Denmark

Contents

1. Text S1 to S4
2. Figures S1 to S10
3. Tables S1 to S2

Table 1. Ice velocity sources. SAR based IV maps: PROMICE: www.promice.dk and ESA CCI Greenland: http://esa-icesheets-greenland-cci.org. The maps were generated using intensity offset tracking. Optical based: Optical feature tracking using ImGRAFT.

Source	Sensor	Time period
ESA CCI Greenland	ERS-1 and 2, Envisat (SAR)	19910826 to 20100507
ESA CCI Greenland	Sentinel-1 (SAR)	20150122 to 20170322
PROMICE	Sentinel-1 (SAR)	20160913 to 20190909
ImGRAFT	Landsat 5-8 (Optical)	19850425 to 20170926

Surface Elevation Change and Slope

In order to investigate changes in surface slope, the discrete measurements of surface elevation from Promice and OIB were interpolated onto the best linear fit to the respective

Corresponding author: A. M. Solgaard, aso@geus.dk

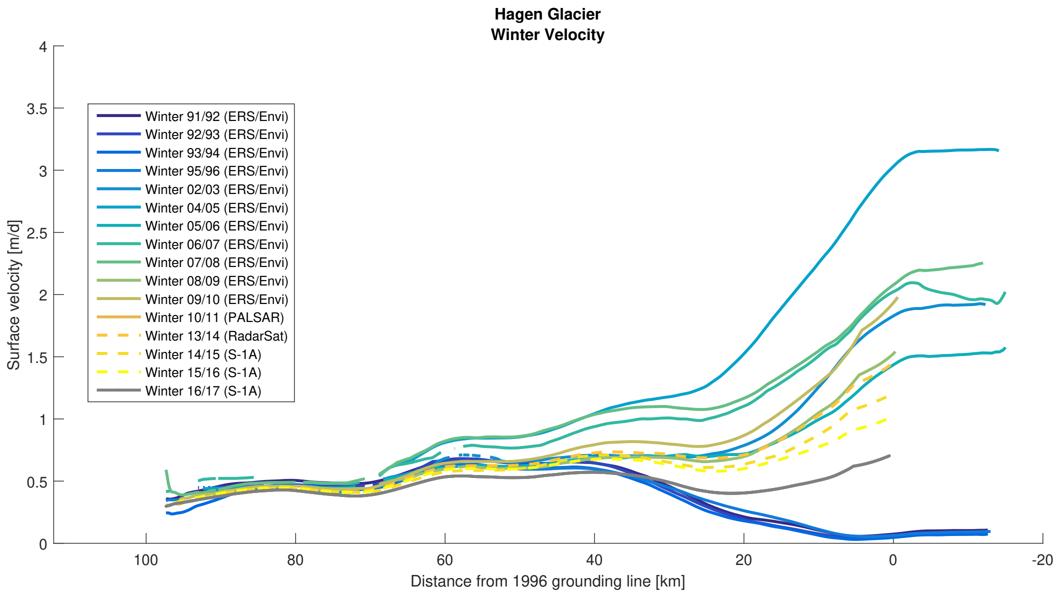

Figure 1. Winter velocities along the main flowline. Data from ESA CCI project and Promice

flight line. The surface elevation was smoothed by a running average over 150 m. The slope along the flight line was then computed by calculating the slope over $\approx$ 3 km (5-6 icethicknesses) (Figure S4 and S5).

Glacier Area Change

The glacier area change was measured by manually digitising the annual glacier front using optical imagery and arcGIS. The origin and resolution of the used imagery is summarised in Table 2. The annual area change was measured from 1985 to 2018 using imagery from Landsat and Sentinel-2. In order to measure the glacier area change consistently the satellite images best representing the annual maximum seasonal retreat were chosen among the available images. Furthermore, only ice free areas (such as large crevasses) with connection to the front line were excluded from the area measurement.

In 2003 the Scan Line Corrector (SLC) on Landsat-7 Enhanced Thematic Mapper Plus (ETM+) failed resulting in bands of missing data in all images *Jensen et al.* [2016]. If the image representing the maximum retreat had missing-data bands crossing the glacier front, the front was digitised as a straight line across the band.

When finding the images, the time resolution of the glacier front was close to daily in the period covered by Landsat 7, Landsat 8 and Sentinel-2, making cloudy weather con-

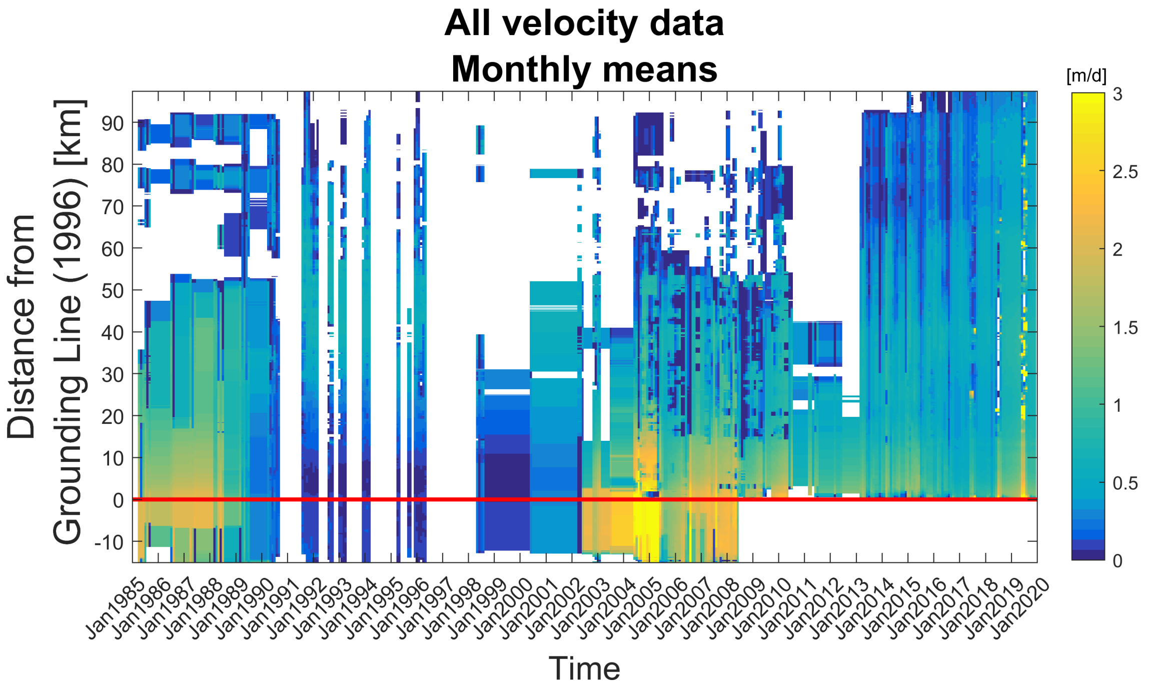

Figure 2. Hovmøller diagram om the average monthly IV along the flowline using all the IV data.

ditions and personal judgment the only possible uncertainty sources when finding the image showing the glacier front position at the end of melt season. Landsat 5 had the same return period as Landsat 7 and Landsat 8 but the number of images available online is much smaller. Thereby, introducing a higher uncertainty as to whether the digitised glacier front represents the front position at the end of melt season correctly. Due to the poor temporal resolution during the first 14 years, the annual glacier area change from this period is associated with a higher uncertainty than the glacier area change later in the period.

The annual glacier area change is also associated with an uncertainty related to human precision and ability to correctly asses the glacier front position, which is often difficult due to shadows and unattached ice floating close to the glacier front. This uncertainty is higher in the measurements from the period before the glacier tongue broke up in the summer of 2008 because the ice bergs had a tendency to stay in the fjord. Thus making it difficult to see which parts of ice were attached to the glacier tongue and which were not. After the tongue broke up in 2008 the tendency for the calved-off ice to stay in the fjord was smaller.

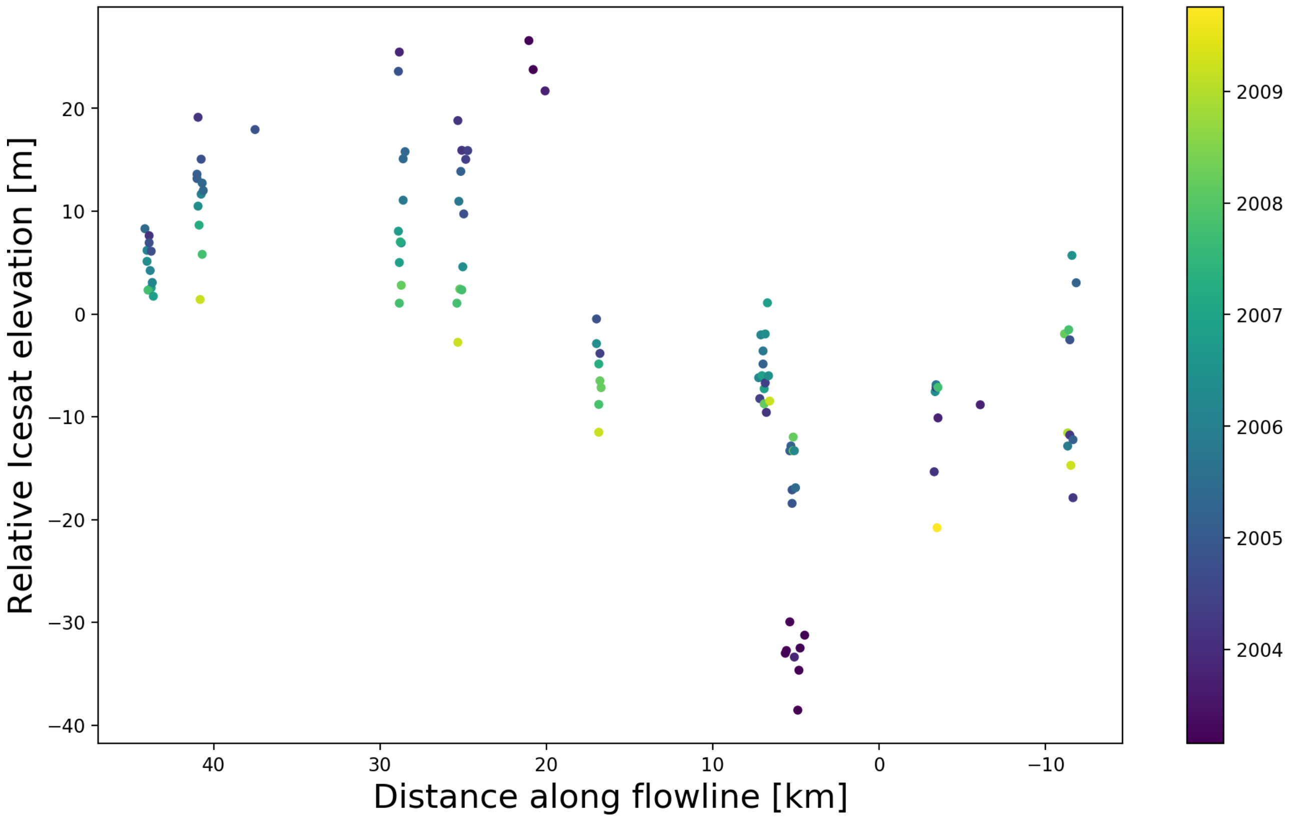

Figure 3. The Hagen flight-line was visited multiple times during the ICESat satellite LIDAR altimeter mission from 2003-2009. Here, the time series of ICESat crossing the flight-line is are presented. The a-cross track slope-dependency of the observations have been removed by subtracting the AreoDEM from 1978. This gives the relative elevation throughout the period 2003-2009 where only limited PROMICE observations are available.

Surface Mass balance Modelling

Using output of surface mass balance from the regional climate model HIRHAM5, we tested whether the increase in surface slope was indeed due to the dynamical behaviour of the glacier or to an increased surface mass balance over the period. Figure S9 was calculated using winter velocities (weighted average of available surface velocity maps between October 1st and March 31st) since 1992. The total surface mass balance of the area between the gates was added to the flux through the upper gate and compared the flux through the lower. There is a slight decreasing trend in the surface mass balance over the period already indicating that it was not the controlling factor in the surface elevation changes. In Figure 9 the difference between the amount of ice going in and out of

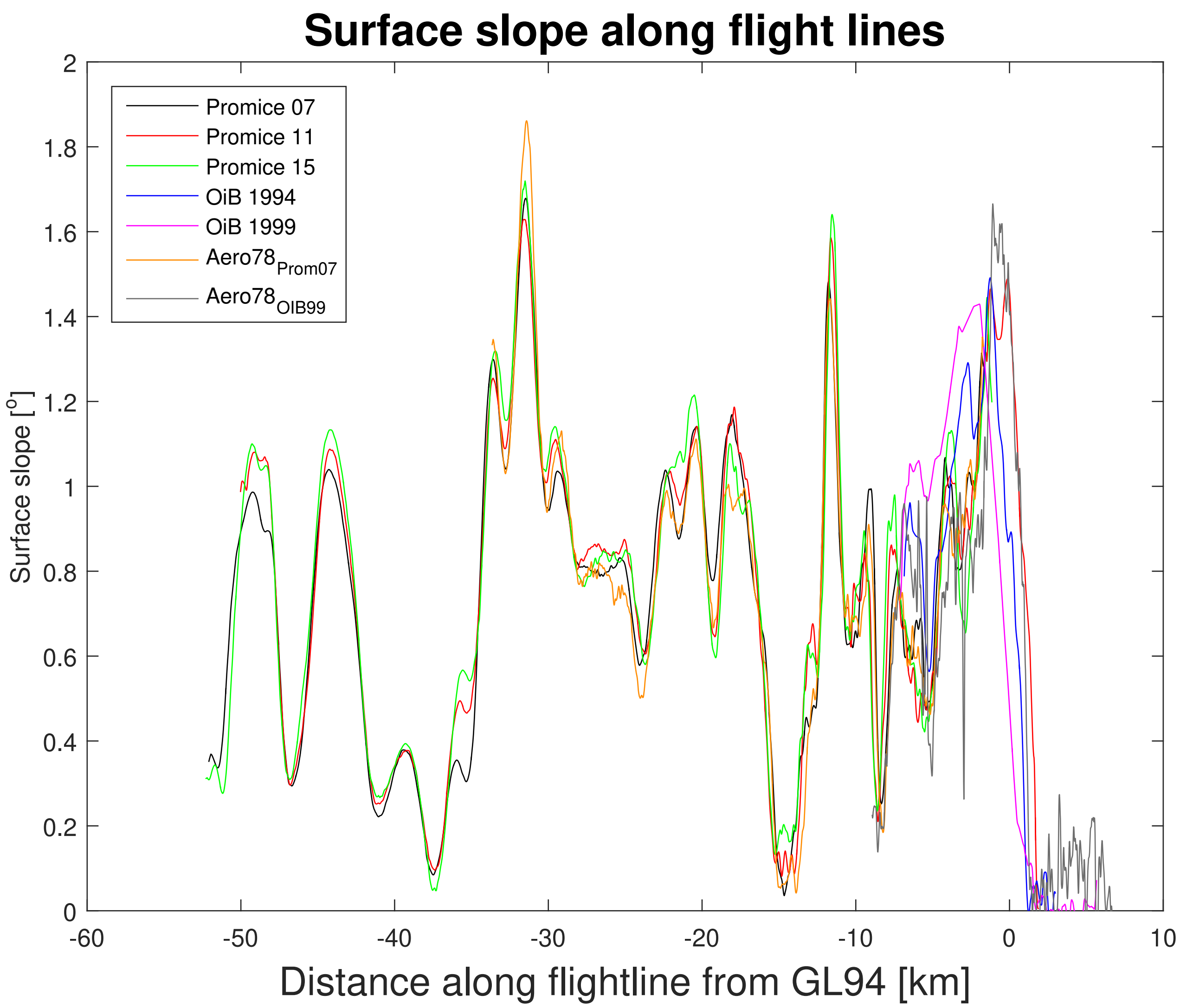

Figure 4. Surface slope along the flightlines and AeroDEM interpolated onto both Promice and OIB flightlines.

the area between the gates distributed evenly over the area is displayed. In the 1990's the result indicates an increase in surface elevation of the order of 1 m/yr while during the peak of the surge the surface lowered by a rate of more than 2 m/yr decreasing to less than 1 m/yr in recent years consistent with observations. The observed changes in surface elevation are thus controlled by the changes in flow and are not due to changes in surface mass balance.

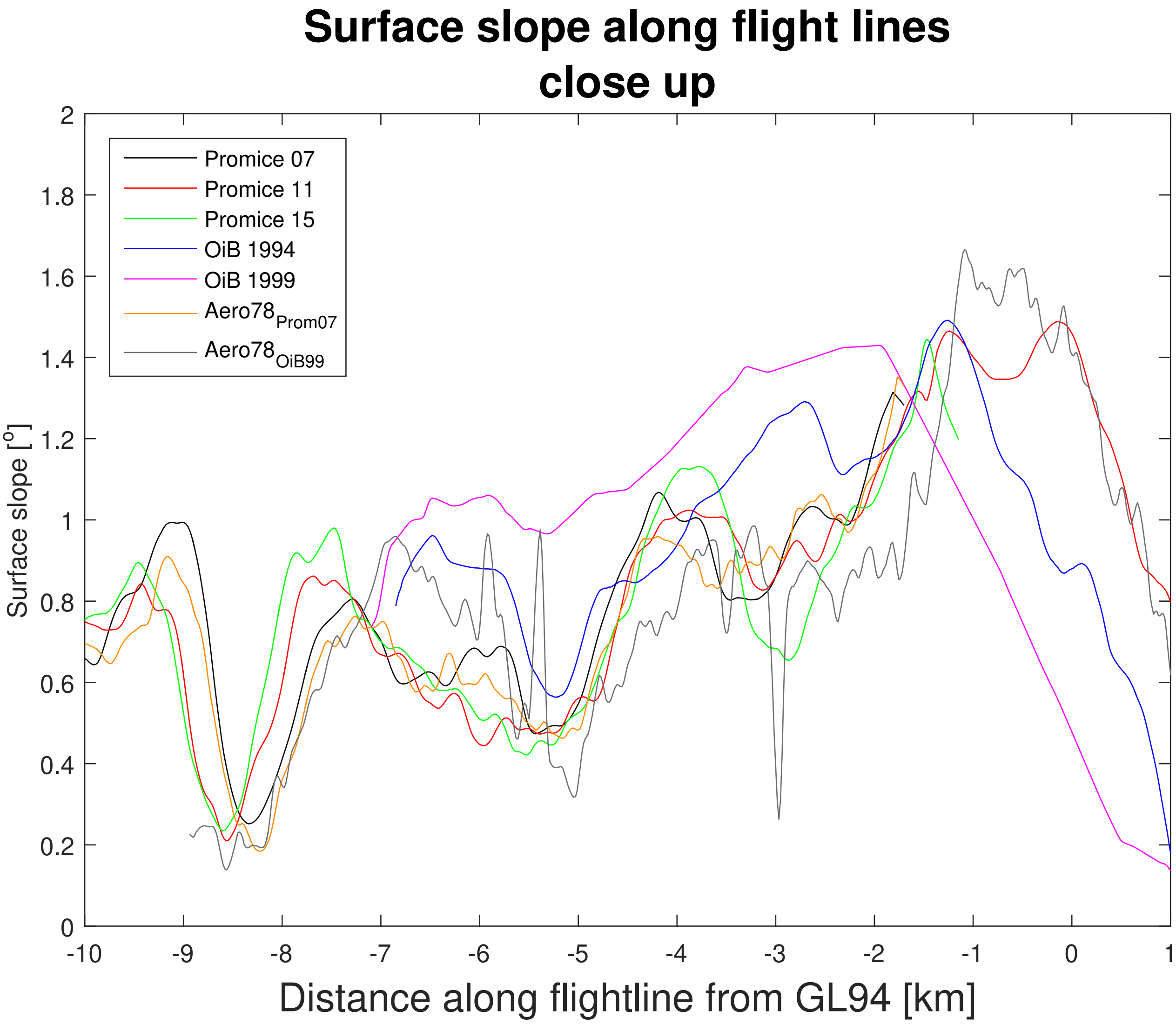

Figure 5. Surface slope: zoom in on the area between grounding line 1994 and 10 km inland in Figure 4.

Break up of Sea Ice in the Fjord in front of the Glacier

Using Landsat and Sentinel-2 images we manually determine the day of the year when the sea ice broke up in front of Hagen Bræ since 1985. The precision of this date is of course dependent on available images and cloud cover. It is not possible to detect sea ice break up for all years due to the temporal coverage of the images.

References

Jensen, T. S., J. E. Box, and C. S. Hvidberg (2016), A sensitivity study of annual area change for Greenland ice sheet marine terminating outlet glaciers: 1999 – 2013, *Jour-*

Table 2. Overview of used satellite imagery

Satellite	Band	Resolution	Years
Landsat 5	3	30 m	1985 - 1998
Landsat 7	3-5	30 m	1999 - 2002 + 2011
Landsat 7	8	15 m	2003 - 2010 + 2012
Landsat 8	4-6	30 m	2013 - 2017
Sentinel-2	8	10 m	2018

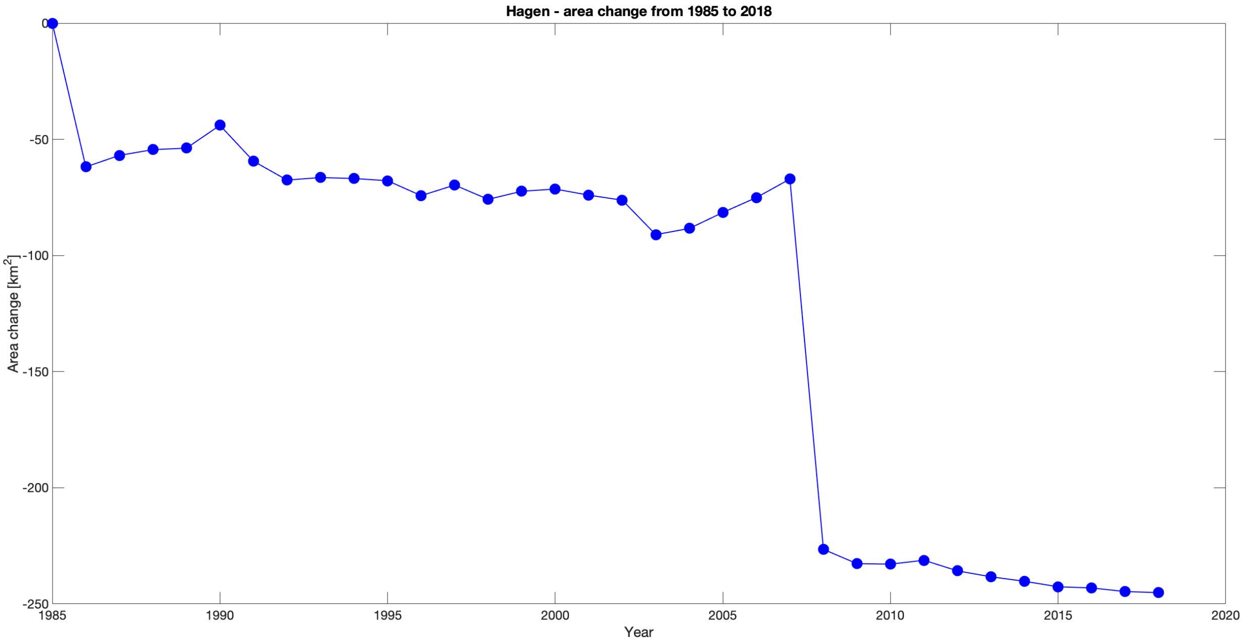

Figure 6. Glacier area change relative to 1985.

nal of Glaciology, *62*(231), 72–81, doi:10.1017/jog.2016.12.

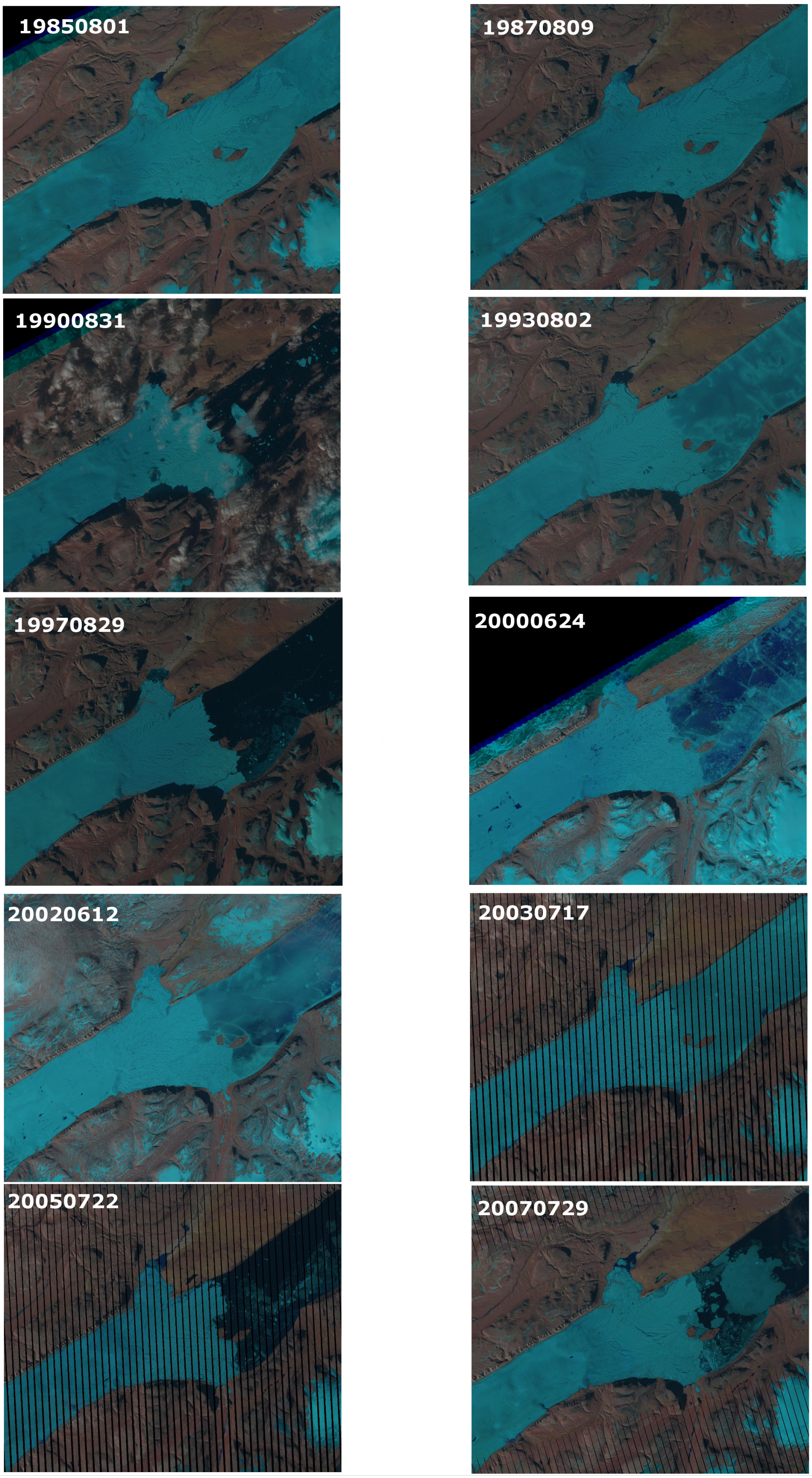

Figure 7. Timeseries of Landsat images showing the evolution of the outer part of the glacier.

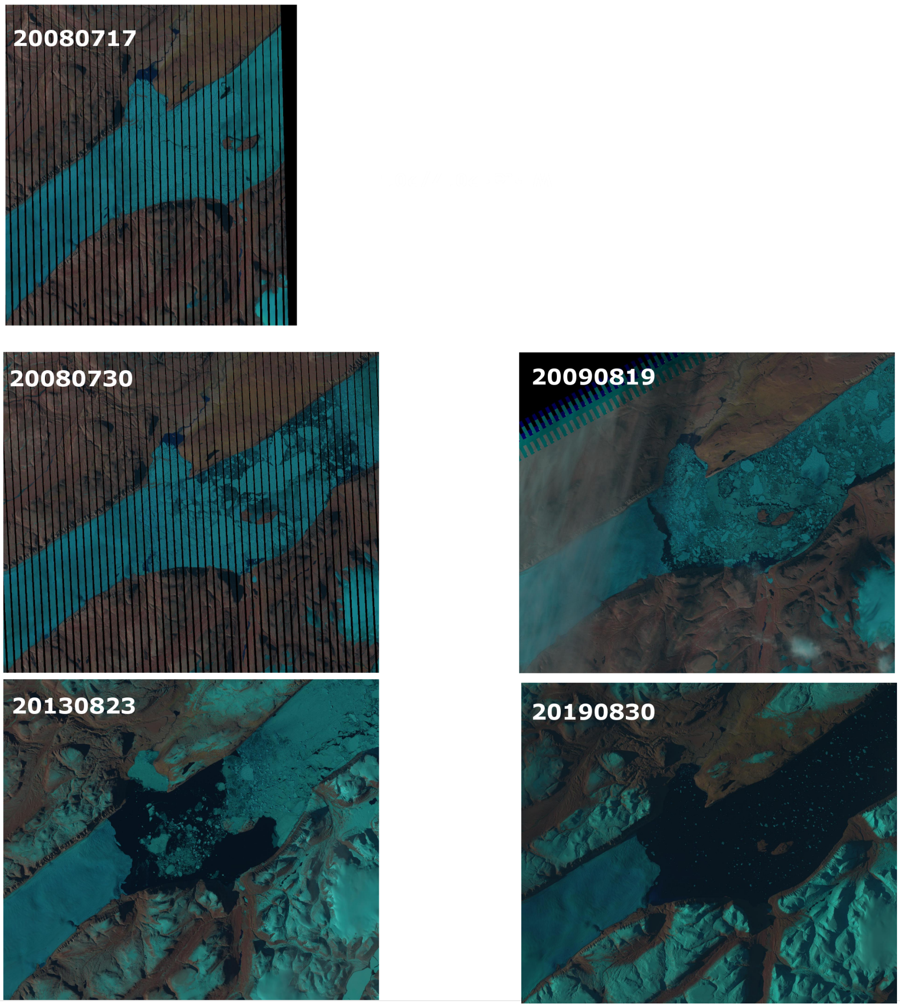

Figure 8. Timeseries of Landsat images showing the evolution of the outer part of the glacier.

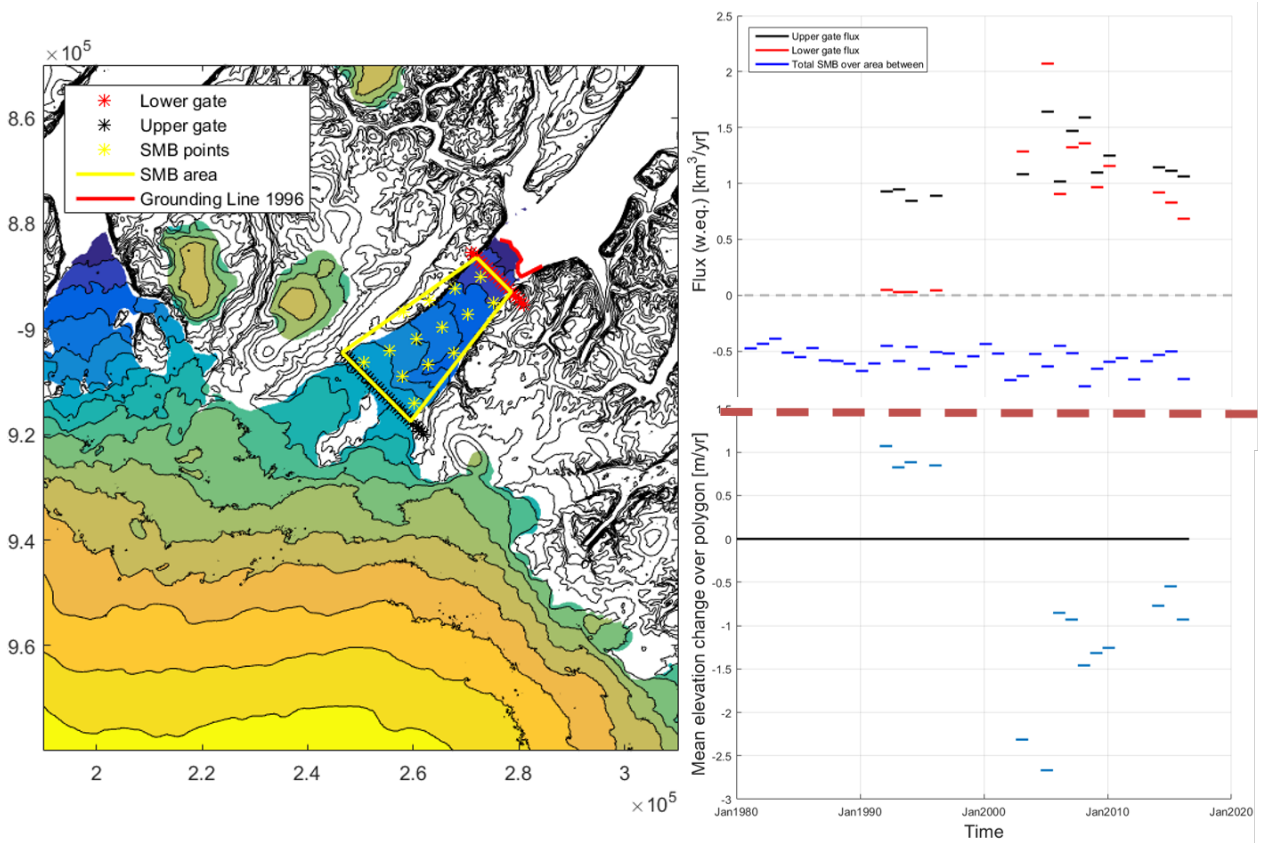

Figure 9. Flux through fluxgates. Left: The location of the upper and lower flux gates are shown on the map indicated by black and red starts, respectively. The yellow polygon indicates the glacier area in between the flux gates where the surface mass loss modelled by HIRHAM5 is calculated. Right: Upper plot shows the ice flux through the gates as a function of time as well as the surface mass balance. Lower: The plot shows average surface elevation change inside the yellow polygon due to the net accumulation/loss each year.

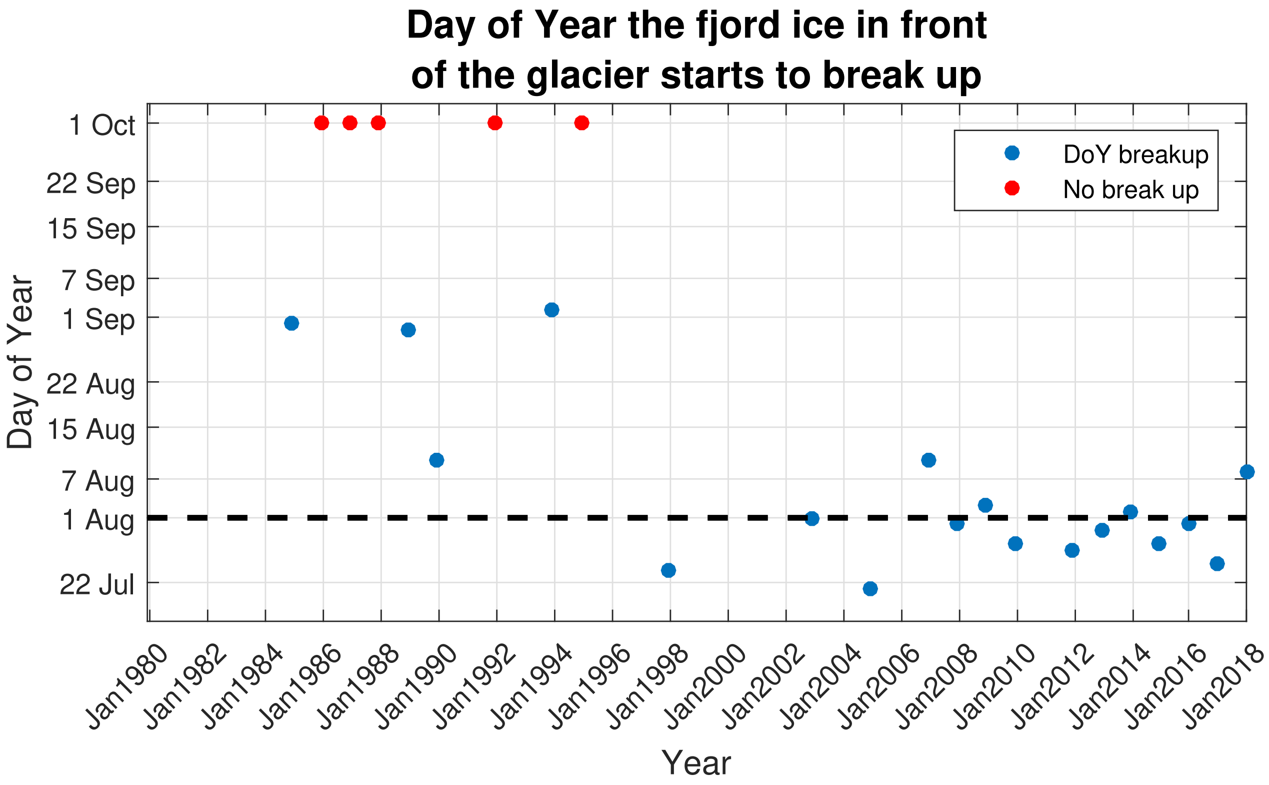

Figure 10. The date for break-up of the sea ice in the fjord in front of Hagen Bræ is found by manual inspection of optical imagery from Landsat and Sentinel-2 and is indicated by blue dots. Red dots indicate that the ice did not break up that particular year.
